# Establishment of Normal Gut Microbiota Is Compromised under Excessive Hygiene Conditions

**DOI:** 10.1371/journal.pone.0028284

**Published:** 2011-12-02

**Authors:** Bettina Schmidt, Imke E. Mulder, Corran C. Musk, Rustam I. Aminov, Marie Lewis, Christopher R. Stokes, Mick Bailey, James I. Prosser, Bhupinder P. Gill, John R. Pluske, Denise Kelly

**Affiliations:** 1 Gut Immunology Group, Rowett Institute of Nutrition and Health, University of Aberdeen, Aberdeen, United Kingdom; 2 Veterinary Pathology, Infection and Immunity, School of Clinical Veterinary Science, University of Bristol, Bristol, United Kingdom; 3 Institute of Biological and Environmental Sciences, University of Aberdeen, Aberdeen, United Kingdom; 4 Agricultural and Horticultural Development Board, Milton Keynes, United Kingdom; 5 School of Veterinary and Biomedical Sciences, Murdoch University, Murdoch, Western Australia, Australia; Charité, Campus Benjamin Franklin, Germany

## Abstract

**Background:**

Early gut colonization events are purported to have a major impact on the incidence of infectious, inflammatory and autoimmune diseases in later life. Hence, factors which influence this process may have important implications for both human and animal health. Previously, we demonstrated strong influences of early-life environment on gut microbiota composition in adult pigs. Here, we sought to further investigate the impact of limiting microbial exposure during early life on the development of the pig gut microbiota.

**Methodology/Principal Findings:**

Outdoor- and indoor-reared animals, exposed to the microbiota in their natural rearing environment for the first two days of life, were transferred to an isolator facility and adult gut microbial diversity was analyzed by 16S rRNA gene sequencing. From a total of 2,196 high-quality 16S rRNA gene sequences, 440 phylotypes were identified in the outdoor group and 431 phylotypes in the indoor group. The majority of clones were assigned to the four phyla Firmicutes (67.5% of all sequences), Proteobacteria (17.7%), Bacteroidetes (13.5%) and to a lesser extent, Actinobacteria (0.1%). Although the initial maternal and environmental microbial inoculum of isolator-reared animals was identical to that of their naturally-reared littermates, the microbial succession and stabilization events reported previously in naturally-reared outdoor animals did not occur. In contrast, the gut microbiota of isolator-reared animals remained highly diverse containing a large number of distinct phylotypes.

**Conclusions/Significance:**

The results documented here indicate that establishment and development of the normal gut microbiota requires continuous microbial exposure during the early stages of life and this process is compromised under conditions of excessive hygiene.

## Introduction

The mammalian gut is colonized by a highly complex, diverse and dynamic microbiota. Although considered sterile during gestation, at delivery the gut is exposed to microbes during passage through the birth canal. ‘Environmental’ bacteria are then ingested from the vagina, feces, skin and the early-life environment [1]. Following birth, bacterial transfer to the neonatal intestine is continuous throughout the suckling and nursing periods. The resulting microbiota is very diverse and reflects the microbial communities associated with the birth and rearing environments, as well as maternal contact [2], [3].

Convergence towards a stable commensal gut microbiota is thought to be established in adult life [4], and although significant temporal variability in the microbiota has recently been documented [5], microbiota composition undoubtedly has life-long consequences for the host [6]. Experimental evidence has highlighted its crucial role in regulating complex mechanisms of host development, lipid metabolism, pathogen response, tissue repair and immune homeostasis [7], [8], [9], [10], [11], [12], [13]. Both host-dependent and host-independent factors affect microbial composition and include host genetics, nutrition, mode of delivery, gestational age, rearing environment and antibiotic exposure [14], [15], [16]. For example, microbial colonization in infants delivered by caesarean section occurs later than in naturally-delivered infants and compositional differences in intestinal microbiota appear to persist throughout life [14], [17]. Rearing environment and exposure to antibiotics also have profound effects on the adult gut mucosa-adherent microbiota and immune development in the pig [18]. Analysis of 16S rRNA gene sequences has revealed major differences in mucosa-adherent microbial diversity in the ileum of adult pigs reared in different environments [18]. The gut microbiota of pigs housed in natural outdoor environments was dominated by Firmicutes, in particular by *Lactobacillus* spp., whereas animals housed under hygienic conditions in indoor environments displayed reduced numbers of lactobacilli and higher numbers of Proteobacteria including potentially pathogenic phylotypes.

The aim of the current study was to elucidate the impact of limiting microbial exposure during development, by maintaining animals in environments of excessive hygiene, on the composition and dynamics of the adult pig microbiota. Piglets were originally colonized in outdoor (extensive) and indoor (intensive) rearing systems with distinct microbial communities and then reared in high-hygiene isolators. Mucosa-associated ileal microbiota was analyzed by comparison of 16S rRNA gene sequences from the two groups.

## Materials and Methods

### Ethics Statement

All animal studies were performed according to the regulations and guidance provided under the UK Home Office Animals (Scientific Procedures) Act 1986. Experimental protocols were approved by the University of Bristol Ethical Review Group and the Home Office under project license number PPL 30/2482.

### Experimental animals and tissue sampling

Five Large White×Landrace sows (*Sus scrofa*) were housed either in an outdoor (extensive, OIs) or an indoor (intensive, InIs) rearing facility. The sows were artificially inseminated by the same boar to minimize genetic variation among the offspring. Two days after birth, two piglets per sow (*N* = 5 per group, ten piglets in total) were transferred to isolators (specific pathogen-free, positive-pressure units supplied with a high-efficiency particulate air (HEPA) filter) through a dunk tank containing 1% w/v bactericidal and virucidal disinfectant solution (Virkon; Antec International Ltd, Sudbury, UK). Up until day 28, the piglets were fed a commercial, bovine milk-formula (Piggimilk; Parnutt Feeds, Sleaford, UK) dispensed by an automated liquid feeding system. From day 29 onwards, all piglets were fed creep feed (Multiwean, SCA NUTRITION Ltd) *ad libitum*. The experiment was performed using two consecutive replicates.

At day 56, all piglets were sacrificed by injection of sodium pentobarbitone (Euthesate, Willows Francis Veterinary Ltd). The ileum, defined as the region corresponding to 75% in length from the pyloric sphincter, was excised. 16S rRNA gene libraries were constructed using bacterial DNA derived from this site as it represents a key region involved in microbial antigen sampling, immune induction and effector activity.

### Mucosal microbiota analysis

Ileal tissue was cut open and contents were removed. Tissue was then washed with ice-cold phosphate buffered saline (PBS) and incubated overnight in ice-cold PBS/0.1% Tween 20 (Sigma-Aldrich Inc., Gillingham, UK) solution with continuous shaking. Detached bacteria were harvested by centrifugation at 10,000× g for 10 min at 4°C. Total DNA from the pellet was isolated using a DNA Spin Kit for Soil® (QBiogene Inc., Cambridge, UK) according to the manufacturer's protocol. PCR amplification of 16S rRNA genes was carried out with the universal primer set S-D-Bact-0008-a-S-20 (5′-AGAGTTTGATCMTGGCTCAG-3′) and S-*-Univ-1492-a-A-19 (5′-ACGGCTACCTTGTTACGACTT-3′) [19]. PCR cycling conditions were: one cycle at 94°C for 5 min, followed by 25 cycles at 94°C for 30 s, 57°C for 30 s, 72°C for 2 min, with a final extension at 72°C for 10 min. PCR products were purified with the Wizard® SV Gel & PCR Clean-up System (Promega, Southampton, UK), cloned into the pCR-4 cloning vector and transformed into *E. coli* TOP 10 chemically-competent cells according to the manufacturer's instructions (TOPO TA Cloning Kit; Invitrogen, Paisley, UK). Recombinant colonies were picked and archived in 96-well plates. Inserts were sequenced at the RINH genomics facility (University of Aberdeen, UK) using the primer set S-*-Univ-0907-a-A-20 (5′CCGTCAATTCATTTGAGTTT-3′) and S-*-Univ-0519-a-A-18 (5′-GWATTACCGCGGCKGCTG-3′) [19]. All clone libraries were constructed under identical conditions to minimize sample-to-sample variation. The methods used here are prone to under-sampling, thus the relative differences in gut bacterial composition between the samples is presented. However, on the basis of the clone number analysed, the data presented strongly suggests that microbial diversity was high in both treatment groups. This data is discussed in the context of diversity analysis of naturally-reared littermates from the same experiment for which clone libraries were adequately sampled [18].

### Sequence alignment and phylogenetic analysis

The 16S rRNA gene reads were assembled using the Lasergene 6 package (DNASTAR Inc.; Infogen Bioinformatics, Broxburn, UK). Assembled sequences were tested for possible chimeras using Chimera Check v2.7 (online analysis at RDP-II website, http://rdp.cme.msu.edu/) and Bellerophon (http://foo.maths.uq.edu.au/~huber/bellerophon.pl [20]). Sequences with no close match in RDP-II were additionally subjected to Basic Local Alignment Search Tool (BLAST) analysis (http://www.ncbi.nlm.nih.gov/BLAST). Chimeric and poor quality sequences were excluded from further phylogenetic analyses.

The resulting 16S rRNA gene sequences were aligned using Multiple Sequence Comparison by Log-Expectation (MUSCLE, http://www.ebi.ac.uk/Tools/muscle) and the alignments were inspected manually. The distance matrix (generated from the multiple sequence alignment) was calculated using the Dnadist application of the Phylogeny Inference Package and Jukes-Cantor distance of 0.01. This stringent phylotype definition at 99% cut-off was used because evidence suggests that bacteria with nearly-identical 16S rRNA gene sequences may represent different phylotypes and species [21].

Rarefaction and collector's curves of observed phylotypes, richness estimates and diversity indices were determined with the DOTUR program (http://schloss.micro.umass.edu/software/dotur.html [22]) using Jukes-Cantor corrected distance matrix. The bias-corrected Chao 1 richness estimator was calculated after 1000 randomizations of sampling without replacement. Collector's curves of observed and estimated (Chao 1 and the abundance-based coverage estimator, ACE) richness were constructed. Diversity values were estimated using the Shannon (H) and Simpson indices (D). The Simpson reciprocal index was calculated as 1/D, and another version of the Simpson diversity index as 1-D. The Good's coverage percentage was calculated with the formula [1−(n/N)]×100, where n is the number of phylotypes in a sample represented by one clone (singletons) and N is the total number of sequences in that sample [23].

A similarity search of the 16S rRNA gene sequences against database entries was performed using the Basic Local Alignment Search Tool (BLAST) program at the National Center for Biotechnology Information website (http://www.ncbi.nlm.nih.gov/BLAST). Sequences were assigned to respective bacterial phylotypes using a >99% sequence similarity criterion.

Phylotype comparisons were made among groups of animals using the Mann-Whitney U test. Multiple comparisons were carried out using the Kruskal-Wallis test, with *P*<0.05 considered statistically significant.

### Nucleotide sequence accession numbers

The nucleotide sequences obtained in this study were submitted to the GenBank database under accession numbers JN882713 to JN884815.

## Results

### Bacterial diversity is elevated in pigs naturally colonized but reared in isolators

**Figure 1:**
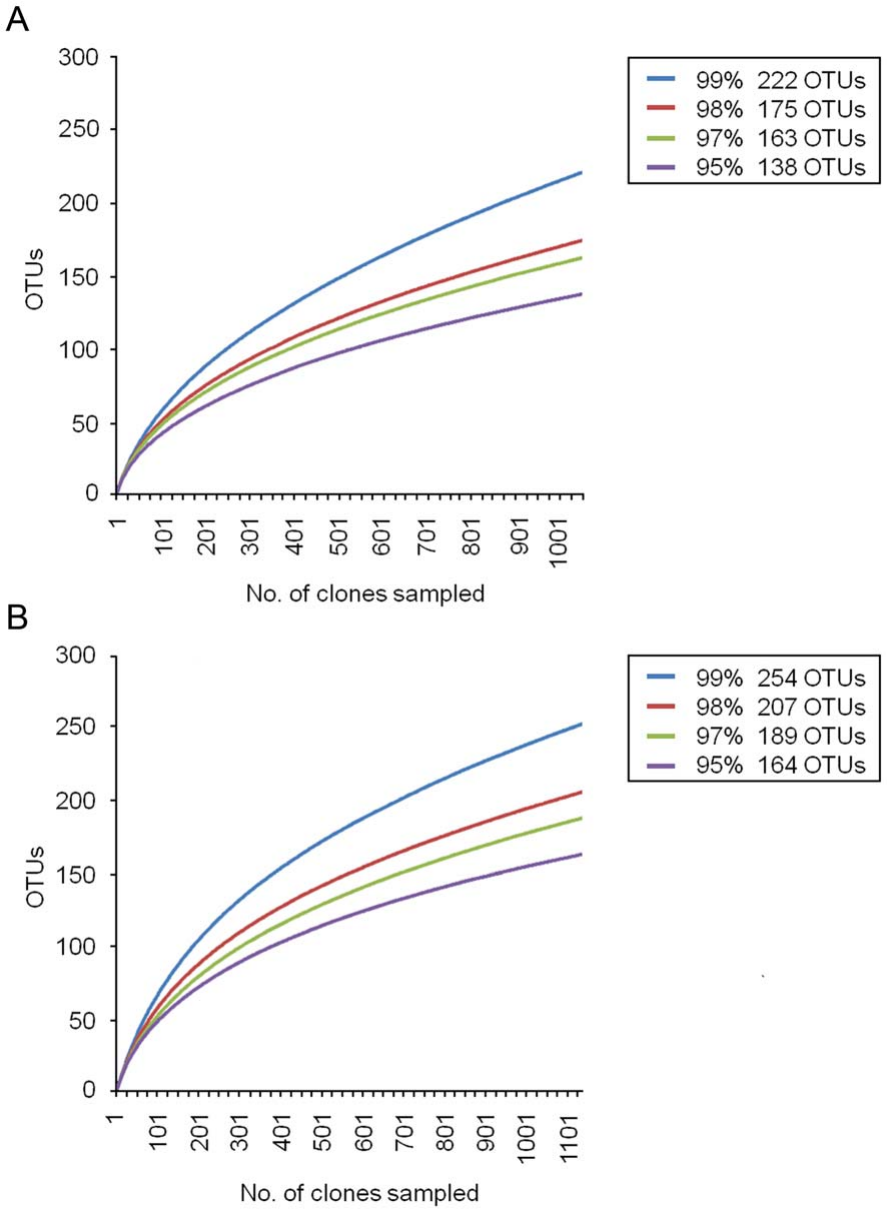
16S rRNA gene library rarefaction curves from isolator-reared animals at multiple OTU cutoff levels. Rarefaction curves were generated by plotting the number of phylotypes (OTUs) against the number of clones sequenced. At 99% cut-off, rarefaction analysis of clone libraries suggested that both the InIs (A) and OIs (B) group possessed a highly diverse mucosa-associated bacterial community. Clearly, even after sampling >1000 clones for each treatment group, the number of OTUs continued to increase even as OTU definitions relaxed towards 95% (genus level).

Rarefaction curves (Fig. 1), which estimates species richness as a function of the number of clones sampled, were generated by plotting the number of phylotypes (operational taxonomic units, OTUs) against the number of newly identified sequences. Neither of the rarefaction curves reached a plateau at the genus (95%) and species levels (99%), indicating that even after sampling over 1000 sequences for each treatment group, the number of OTUs was likely to increase with additional sampling. Rarefaction curves of the individual animals within the treatment groups are illustrated in Fig. S1 and S2. Interestingly, within both treatment groups the individual diversity varied greatly between animals, suggesting strong genotypic influences. The high number of OTUs encountered in some individuals contributed to the overall high diversity observed in both treatment groups. Additional sampling is required to determine the true gut bacterial diversity in these adult animals, but the data presented strongly suggests that the microbial diversity is high in both treatment groups.

**Figure 2:**
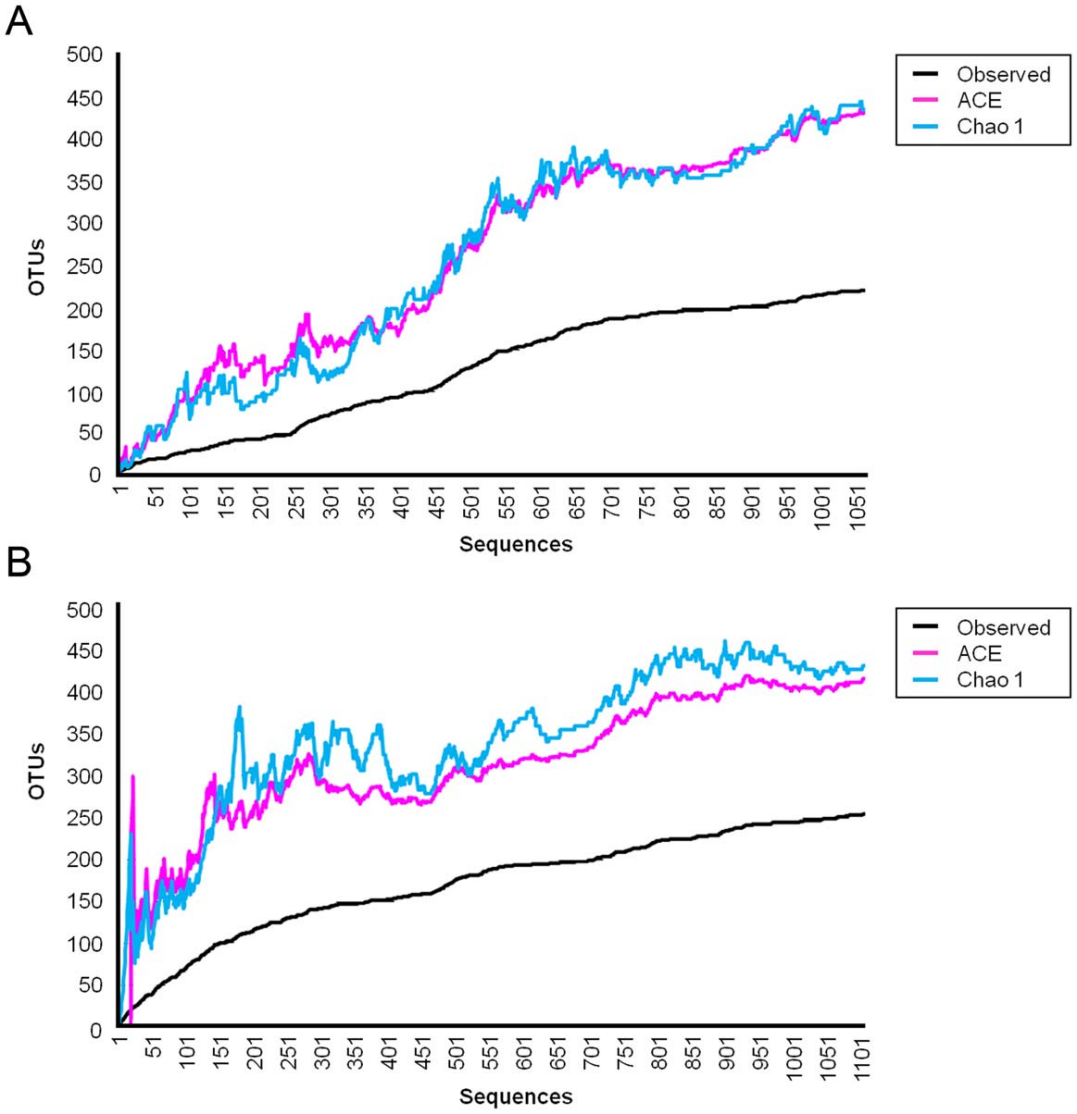
16S rRNA gene library collector's curves from indoor isolator-reared and outdoor isolator-reared animals. Collector's curves of the observed (black) and estimated (Chao1 (blue) and ACE (pink)) phylotype richness calculated at 99% OTU cut-off level from indoor isolator-reared (A) and outdoor isolator-reared animals (B). The relatively constant estimates of the number of unobserved phylotypes in each treatment group as observed richness increases indicate that estimated richness is likely to increase further with additional sampling.

Collector's curves were constructed as plots of the cumulative number of OTUs recorded as a function of sampling effort (number of clones sampled from each clone library). Sequences with a similarity >99% were considered to belong to the same OTU. Collector's curves of the observed and estimated phylotype richness are shown in Fig. 2. Each curve reflects the series of observed or estimated richness values obtained as clones were added to the data set in a random order. The curves rose less steeply as a decreasing proportion of new phylotypes were encountered in the treatment groups. The number of unseen phylotypes was represented by the gap between the observed phylotypes and the number of phylotypes estimated by Chao1 and ACE. The difference between the estimated and observed phylotype richness was high in both isolator treatment groups. Novel phylotypes continued to be identified throughout sampling. The relatively constant estimates of the number of unobserved phylotypes in each treatment group as observed richness increased indicated that the estimated richness was likely to increase further with additional sampling. The overall species richness in the OIs group was estimated at 440 phylotypes by Chao1 and 438 by ACE (Table 1 and Fig. 2). Estimated phylotype richness was slightly lower in the InIs group with 431 phylotypes as estimated by Chao1 and 416 phylotypes by ACE. Good's coverage was 89.5% for the OIs sequence set and 89% for the InIs sequence set, indicating that eleven additional phylotypes would be expected for every 100 additional sequenced clones. This contrasts with the lower diversity values previously reported for naturally-reared littermates from the same study [18] (Table 1). Compared to their isolator-reared littermates, naturally-reared OUT and IN animals had Chao1 values of 254.9 and 259 and ACE values of 208.1 and 280.2, respectively. The lower diversity in these animals was also reflected in the library coverage, as both libraries had a Good's coverage of greater than 92%. Sequences were subjected to BLAST searches against GenBank entries to assign them to the lower taxonomic ranks.

**Table 1:** Indices of diversity, richness and library coverage for 16S rRNA gene libraries *(N = 5)*.

Measurement	InIs	OIs	IN ^[18]^	OUT ^[18]^
Chao1 estimator of species richness	431.2	440.9	259.0	254
ACE abundance estimator	416.2	438.5	280.2	208
Shannon diversity index (H)	4.5	4.9	4.4	4.2
Simpson diversity index (1-D)	0.98	0.99	0.98	0.97
Simpson reciprocal index (1/D)	44.6	89.6	52.0	40.4
Good's estimator of coverage (%)	89%	89.5%	93.5	92.5

Calculations were made based on OTU definition at 99% sequence identity.

### Taxonomic placement of sequences into 4 major phyla

The 16S rRNA genes from the mucosa-associated ileal samples were subjected to the RDP Classifier analysis (with a 95% confidence threshold). Based on the classification results, the clone sequences were assigned to four phyla: Firmicutes (67.5% of all sequences), Proteobacteria (17.7%), Bacteroidetes (13.5%), and Actinobacteria (0.1%) (Table 2). 1.2% of the sequences remained unclassified by the RDP Classifier. These results largely correspond to the distribution across the bacterial phyla in the naturally-reared animals (OUT and IN), as previously reported [18], where clones were assigned to Firmicutes (69.7% of all sequences), Proteobacteria (17.7%), Bacteroidetes (11.4%), and Actinobacteria (0.5%). However, when comparing outdoor sow-reared and outdoor-isolator reared animals directly, an increase in Bacteroidetes from 1.08% to 16.5% and Proteobacteria from 4.63% to 19.5%, coinciding with a reduction in Firmicutes from 94% to 62.5%, was noted (Table 2). Within the Firmicutes, the Lactobacillales were the most affected taxon with a reduction from 81.6% to 15.2%. When comparing indoor sow-reared and indoor-isolator reared animals, an increase in Bacteroidetes (3.72% to 10.2%) and a decrease in Proteobacteria (28.26% to 15.7%) was observed.

**Table 2:** Taxonomic composition of the mucosa-associated microbiota of indoor and outdoor isolator-reared animals and their sow-reared counterparts.

Phylum	Bacterial taxa	InIs	OIs	IN^[18]^	OUT^[18]^
**% ** ***Bacteroidetes***		**10.2**	**16.5**	**3.72**	**1.08**
	Family *Prevotellaceae* (%)	9.5	13.0	2.91	0.54
	Family *Bacteroidaceae* (%)	0.7	0.2	0.40	0
	Family *Porphyromonadaceae* (%)	0	2.9	0	0.40
**% ** ***Proteobacteria***		**15.7**	**19.5**	**28.26**	**4.63**
	**Class α-proteobacteria (%)**	**0.3**	**0**	**0**	**0.13**
	**Class β-proteobacteria (%)**	**0**	**0**	**0.20**	**0**
	**Class γ-proteobacteria (%)**	**15.4**	**19.5**	**15.19**	**3.81**
	Family *Pasteurellaceae* (%)	9.1	14.9	14.78	2.17
	Family *Enterobacteriaceae* (%)	5.9	4.2	0.4	1.36
	Family *Pseudomonadaceae* (%)	0	0.3	0	0
	Family *Moraxellaceae* (%)	0.2	0	0	0
	**Class ε-proteobacteria (%)**	**0**	**0**	**12.97**	**0.4**
	Family *Helicobacteraceae* (%)	0	0	10.46	0.4
	Family *Campylobacteraceae* (%)	0.1	0	2.61	0
**% Firmicutes**		**73.1**	**62.5**	**66.29**	**94.0**
	**Class Erysipelotrichi (%)**	**1.2**	**0.8**	**0.90**	**0**
	**Class Bacilli (%)**	**42.8**	**15.2**	**18.81**	**81.8**
	Order Bacillales (%)	0.6	0	0.2	0.2
	Order Lactobacillales (%)	42.2	15.2	18.6	81.6
	**Class Clostridia (%)**	**28.7**	**46.5**	**46.68**	**12.12**
	Family *Lachnospiraceae* (%)	17.4	23.4	3.52	0.95
	Family *Veillonellaceae* (%)	1.0	1.1	0	0.4
	Family *Clostridiaceae* (%)	2.8	2.3	13.17	2.72
	Family *Peptostreptococcaceae* (%)	3.9	9.7	24.44	7.49
	Family *Ruminococcaceae* (%)	2.7	8.8	0.90	0.54

#### Firmicutes

67.5% of all sequences (1161 clones) were affiliated with the Firmicutes phylum. Bacilli (41.8%) and Clostridia (56.5%) were the most abundant bacterial classes within this phylum, with Erysipelotrichi (1.5%) in low abundance (Table 2).

**Figure 3:**
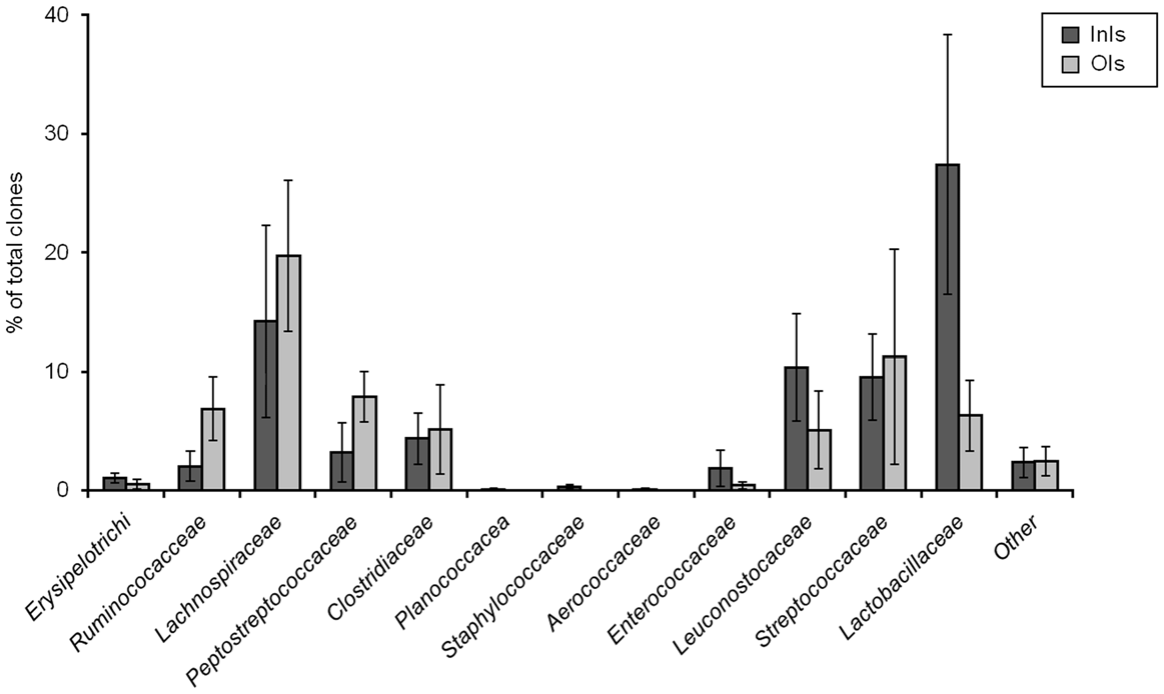
Phylogenetic distribution and abundance of the *Firmicutes* phylum in the mucosa-associated microbiota from isolator-reared animals (*N* = 5).

The most abundant order in the Bacilli class was Lactobacillales (480 clones), including *Lactobacillacaeae*, *Leuconostocaceae*, *Streptococcaceae* and, in lower abundance, *Enterococcaceae* and *Aerococcaceae* (Fig. 3).

**Figure 4:**
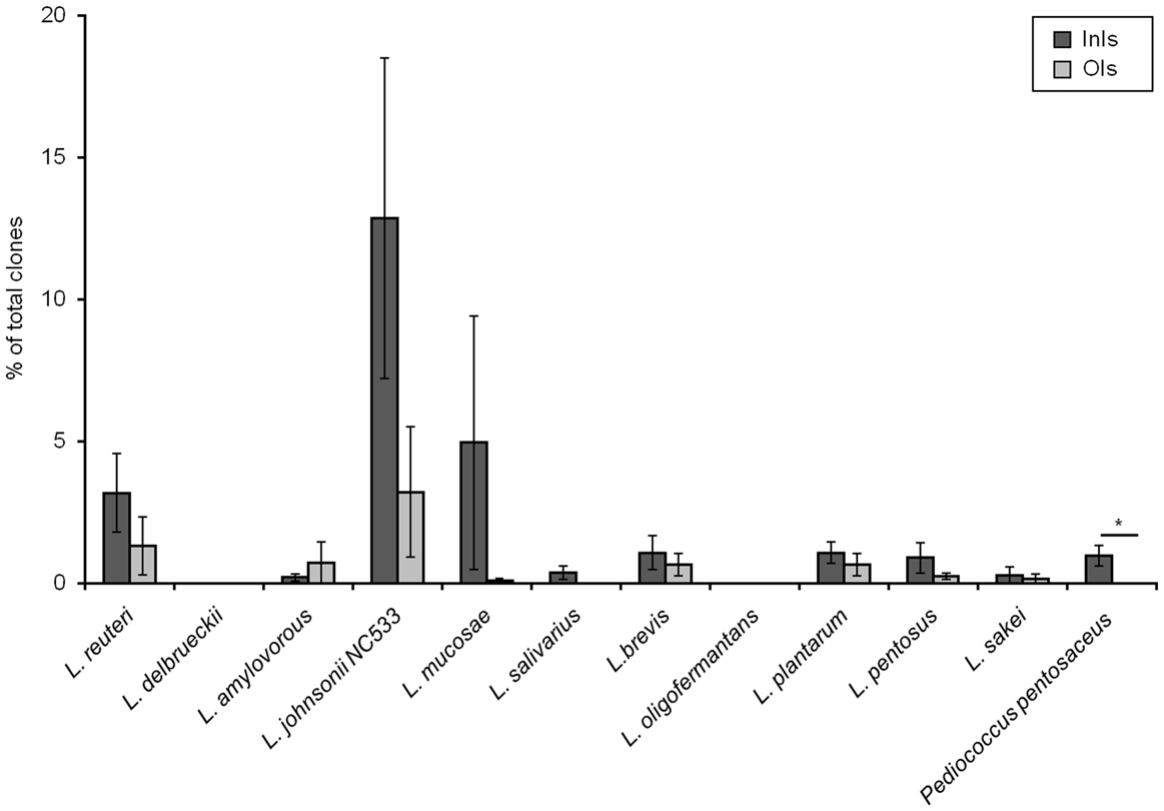
Abundance of *Lactobacillaceae* in the mucosa-adherent microbiota of isolator-reared animals (*N* = 5).

The *Lactobacillaceae* family in the OIs group (7.8% of OIs sequences) consisted of only a small number of OTUs, including *Lactobacillus reuteri*, *L. amylovorous*, *L. johnsonii*, *L. brevis*, *L. pentosus* and *L. plantarum*. The InIs library contained 27.4% *Lactobacillaceae*-affiliated clones, with similar phylotypes to the OIs group including *L. reuteri*, *L. amylovorous*, *L. johnsonii*, *L. brevis*, *L. pentosus* and *L. plantarum*. An additional phylotype, not detected in the OIs group, was *Pediococcus pentosaceus* (CP000422) (Fig. 4).

*Leuconostocaceae* were represented by a total of 150 clones, 101 of which were obtained from the InIs group and the remaining from the OIs group. Within the InIs group, three OTUs were present. All sequences had 99% similarity to *Weissella paramesenteroides* (AB362621), *W. hellenica* (AB015642) and, in lower abundance, *W. cibaria* (AB362617). Interestingly, in the OIs group only animals in replicate 1 possessed members of the *Leuconostocaceae* family. Similar phylotypes were obtained from the OIs group including *W. hellenica* (AB015642) and two phylotypes related to *W. paramesenteroides* (AB362621). *W. paramesenteroides* strain CTSPL5 (EU855224) was the predominant phylotype with 27 clones. None of these phylotypes showed a significant difference between the two treatment groups.

*Streptococcaceae* were represented in lower abundance with a total of 87 clones, 70 of which were detected in the InIs group and the remaining in the OIs group. Strains belonging to *Streptococcus suis* (AF009481), *Str. thermophilus* strain Y-2 (DQ911624), *Str. parauberis* (FJ009631) and *Lactococcus lactis* subsp. lactic (AE005176) were identified. Uncultured bacterium clone SQ_aah81g09, which possessed 99% similarity to *Str. gallolyticus*, was found in high abundance in the InIs group (22 clones). Both *Str. suis* and *Str. gallolyticus* have been implicated in a wide variety of infections in pigs including pneumonia and septicemia [24], [25] and have also been described as human pathogens [26].

From 20 *Enterococcaceae*-related clones, 18 were detected in the InIs group. *Enterococcus gallinarum* strain 22B (EF025908) was the most abundant phylotype. *E. gallinarum*, a motile bacterium, is primarily found in the gastrointestinal tract and in food products [27] and plays a role in invasive infections in humans, especially in immune-compromised or chronically ill patients [28], [29], [30]. Four additional OTUs were identified as *Enterococcus* sp. DJF_O30 (EU728749), *E. avium* (AF133535), *E. faecalis* (AB362601) and *E. italicus* strain 1102 (EF535230). Two sequences related to *E. faecalis* and *E. italicus* were identified in the OIs group. Due to high variation between animals in the treatment groups, no statistically significant difference in the proportion of Enterococci was observed.

#### Clostridia

Members of the Clostridia class were present in all treatment groups (Table 2). The OIs group showed a higher abundance of this class. A total of 353 clones were grouped into *Lachnospiraceae*, with 140 clones obtained from the InIs group and the remaining from the OIs group (Fig. 3). The *Lachnospiraceae* family was represented by a large number of OTUs, mainly uncultured clones. Sixteen distinct OTUs had less than 97% sequence similarity to database entries. The most abundant members obtained from the InIs group included uncultured bacterium clone p-2176-s59-3 (AF371605), *Eubacteriaceae* bacterium DJF_CR57k1 (EU728737) and clone p-2482-18B5 (AF371541). Similar phylotypes were detected in the OIs group in equal numbers. The *Peptostreptococcaceae* family was another abundant member of the Clostridia class, accounting for 7% of the sequenced clones (120 sequences). Thirty-two clones originated from the InIs group (3.9% of InIs sequences) and 88 clones from the OIs group (9.7% of OIs sequences). In the InIs group, 32 clones were obtained exclusively from replicate 1 animals. One predominant phylotype had 99% identity to uncultured bacterium clone VWP_aaa01b10 (EU475070), isolated from the feces of Visayan warty pigs [31]. This clone was also found in high abundance in the OIs group. Interestingly, this clone was only recovered in high abundance in animals from replicate 1 and was not obtained from replicate 2 animals. Replicate 2 animals shared similar phylotypes distinct from replicate 1 animals including uncultured bacterium clone BARB_aaa01h10 (EU475654) and uncultured bacterium clone aaa02d03 (EU475689). Six percent of all sequences were grouped into *Ruminococcaceae* and were mainly represented by a diverse range of uncultured clones. The most abundant clones included uncultured bacterium clone SJTU_C_03_14 (EF403979), which was also obtained from the OIs group, and uncultured bacterium clone p-2609-9F5 (AF371720) in the OIs group. Eighteen clones were affiliated with the *Veillonellaceae* family, including clone VWP_aaa01c05 (EU779292) in the InIs group and clone D19 (AM500725) in the OIs group. Thirty-nine clones belonging to the *Clostridiaceae* family were obtained from both groups and were mainly represented by two clones including VWP_aaa03f12 (EU779318) and RL179_aao56e08 (DQ796928). For all three bacterial families (*Veillonellaceae*, *Clostridiaceae* and *Ruminococcaceae*) the majority of clones were obtained from replicate 1 animals in both treatment groups.

#### Bacteroidetes

**Figure 5:**
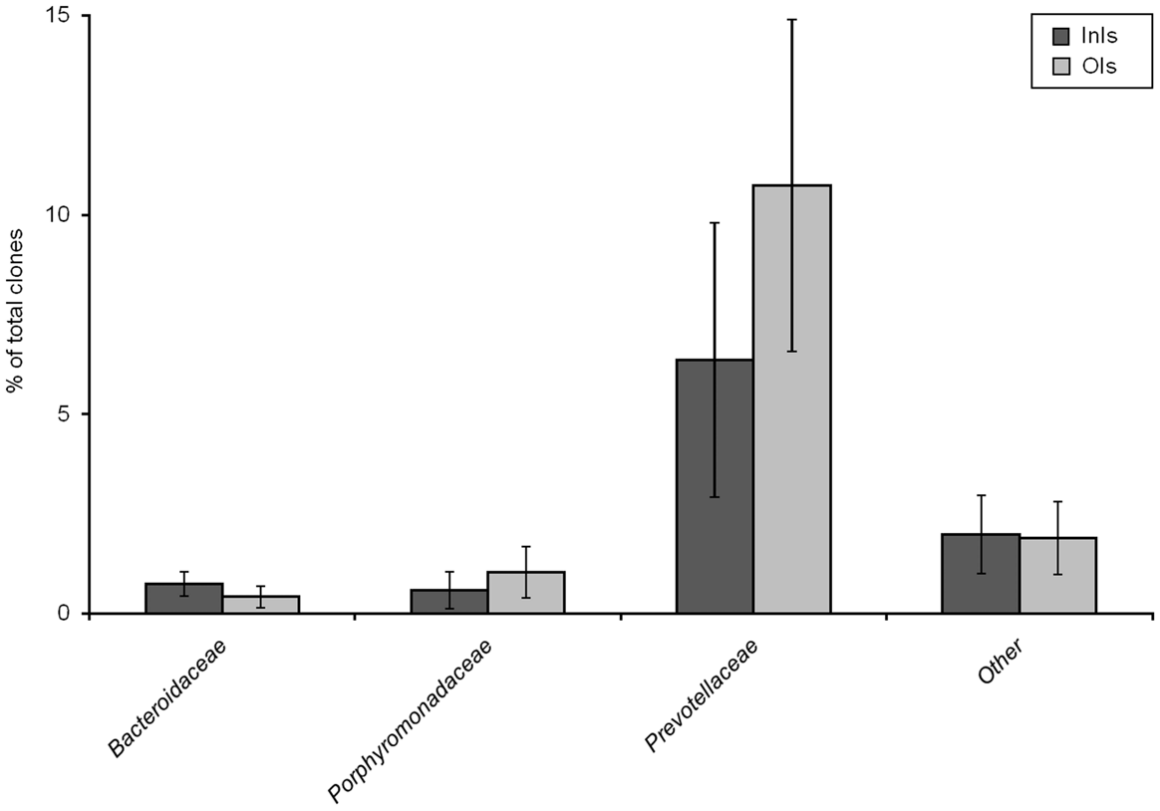
Phylogenetic distribution and abundance of *Bacteroidetes* in the mucosa-associated microbiota of isolator-reared animals (*N* = 5).

All 16S rRNA gene libraries contained sequences related to *Prevotellaceae*, yet they were most prevalent in the OIs group (Fig. 5). Thirteen OTUs encountered had less than 97% similarity to database entries. Abundant members were identified in both libraries, independent from the farm origin, including uncultured bacterium clone p-1980-s959-5 (AF371890), uncultured bacterium clone SPIM_d08_1 (EU467242), uncultured bacterium clone p-2190-s959-3 (AF371875) and uncultured bacterium clones ML_aaj26e06 and ML_aae88g11 (EU776786/ EU467242). Thirty-six additional OTUs were in low abundance, all with a close similarity to uncultured bacterial clones, thereby contributing to the high diversity in the isolator-reared animals. The 19 clones classified as *Bacteroidaceae* were obtained from both treatment groups (Fig. 5). Fifteen clones were retrieved from the InIs libraries and four sequences from the OIs libraries. In the InIs libraries, eight OTUs were present related to *Bacteroides vulgatus* (CP000139) and *B. plebius* (AB200217). Fourteen clones affiliated with *Porphyromonadaceae* were obtained exclusively from the OIs libraries from two animals in the same replicate (Fig. 5). All 14 clones had only 97% similarity or less to previously isolated clones.

#### Proteobacteria

**Figure 6:**
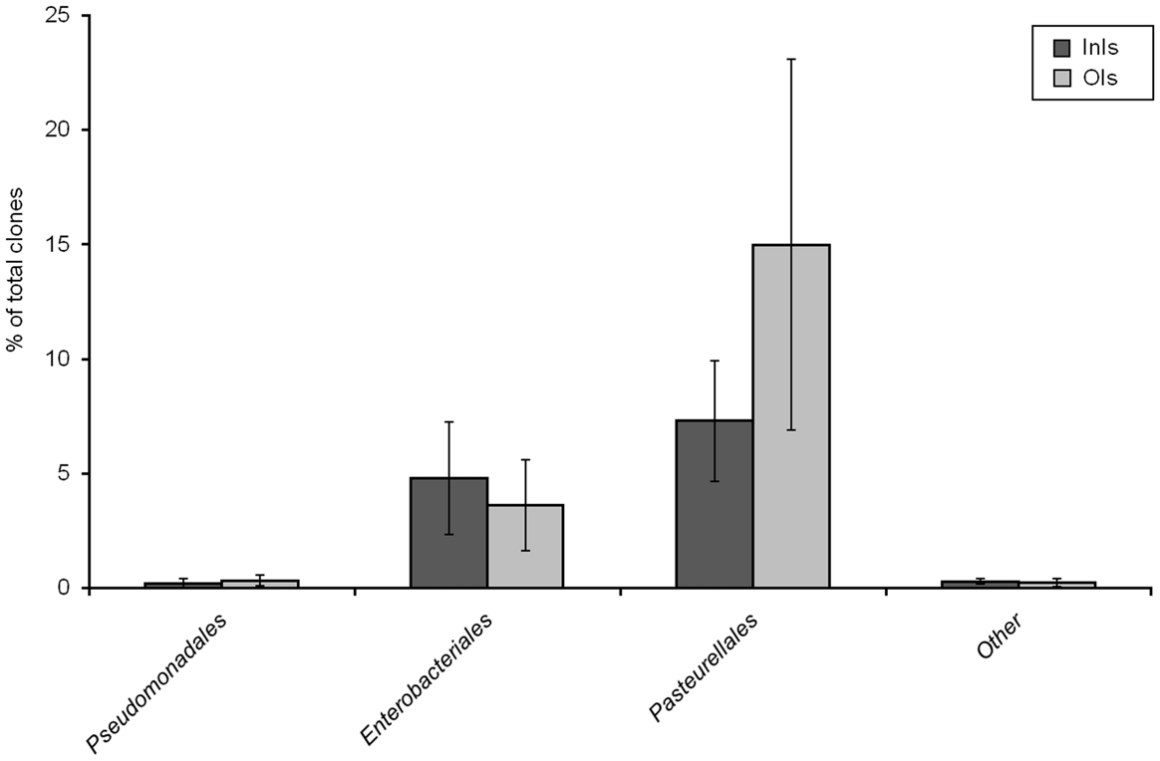
Phylogenetic distribution and abundance of *Proteobacteria* in the mucosa-associated microbiota of isolator-reared animals (*N* = 5).

Overall, 17.7% of the clones (304 sequences) were placed into Proteobacteria, with γ-proteobacteria (302 clones) being the most abundant group. Most of the clones classified as *Pasteurellaceae* (Table 2 and Fig. 6). 177 clones were obtained from the OIs and 127 clones from the InIs mucosa-adherent libraries. Phylotype distribution was very similar between the two treatment groups. The majority of sequences were affiliated with *Actinobacillus minor*, *A. porcinus* and the low abundance sequences with *A. rossii*. This clone has been isolated from the intestine and reproductive tract of pigs and is considered as an opportunistic pathogen implicated in spontaneous abortion. Within the *Enterobacteriaceae* family the most abundant clones were identified as *E. coli* spp.

## Discussion

The mucosal immune system of the pig undergoes rapid changes during the neonatal period, similar to humans [32], [33]. Hence, the pig is increasingly utilized as a translational model [34], [35], [36]. We sought to evaluate the impact of limiting microbial exposure during development on the composition of the pig gut microbiota. Animals were naturally colonized during the first two days after birth and then reared in isolators maintained to a very high-hygiene status. Initially, all piglets remained with the sows, housed in either indoor or outdoor environments, to promote ‘natural’ (environmental and maternal) microbial colonization and to ensure adequate colostrum intake during the first few days of life.

Intriguingly, the 16S rRNA gene sequences generated from adult isolator-reared pigs initially colonized during the early days of life revealed a highly diverse microbiota which included some well-known members of the mammalian gastrointestinal tract as well as previously uncultured phylotypes. Although not well described in the literature, the high sequence numbers and variability within the *Ruminococcaceae*, *Lachnospiraceae* and *Peptostreptoccocaceae* families certainly contributed to the overall microbial diversity of these animals. As a general concept, high microbial diversity is thought to be beneficial to the ecosystem by reducing the opportunity for colonization by infectious agents [37]. In the gut ecosystem, for example, such rich diversity would yield a broad range of immune-triggering compounds required to promote the development of the mucosal immune system [38]. At the early stages of microbial colonization/succession, high microbial diversity, largely reflecting the birth environment, is to be expected as there are few barriers limiting entry of bacteria into the gut ecosystem [39]. However, development of the normal pig microbiota coincides with a natural stabilization of the gut bacterial populations [4], [16], [35], [40], [41] imposed by the strict environmental selection pressures operating within the gut ecosystem. In agreement with this, we previously showed that continuous outdoor environmental exposure in a highly diverse ecosystem resulted in a stable gut microbiota with a lower diversity than in indoor, intensively-reared and isolator-reared littermates [18]. The increased mucosal diversity in isolator-reared animals would therefore suggest that environmental and immune-related control of the mucosa-adherent microbiota was reduced by isolation of these animals and succession to the normal stable microbiota was not achieved, with the microbiota remaining more chaotic. Furthermore, despite the fact that the animals originated from distinct microbial rearing environments, no significant differences in the overall microbial composition were observed, although this may also reflect the need for additional sequence information.

In terms of species composition, previous studies have shown that the intestine of neonatal piglets is initially colonized by large numbers of *E. coli* and *Streptococcus* spp. [42]. Generally, *Enterobacteriaceae* are considered to be the early colonizers of the gut [43], [44] and are associated with the mucus layer [45]. *Streptococcus* spp. were also identified in the microbiota of isolator-reared treatment groups in the current study, but Enterobacteria were only rarely recovered.

*L. reuteri*, *L. amylovorous*, *L. johnsonii*, *L. brevis*, *L. pentosus* and *L. plantarum* were found in isolator-reared animals although they were present in lower numbers relative to littermates reared-outdoors [18]. Leser *et al.* [46] reported a similar range of phylotypes associated with the ileum in pigs from different rearing environments. Furthermore, developmental shifts in the dominant lactobacilli species in the pig gut have also been documented [47]. Hence, certain species of lactobacilli may be better adapted to the gut at the various developmental stages and following dietary change. In the current study, the lactobacilli acquired at the very early life stages may not have been sufficiently adapted to the post-weaning gut environment and isolator-reared animals were restricted in their opportunity to acquire other, more adapted species, unlike their outdoor-reared littermates.

Taken together, the experimental evidence presented in this study illustrates that development in environments of excessive hygiene hinders the progression towards an adult-type gut microbiota, despite the acquisition of a highly diverse microbiota in early life. In particular, we noted that Firmicutes were reduced in isolator-reared animals when compared to outdoor-reared littermates. Conversely, Bacteroidetes and Proteobacteria were increased in isolator conditions. This identifies early life as a crucial developmental period during which continual exposure to environmental microbes is required to drive the ‘stabilization’ of the gut microbiota towards an adult phenotype. Consistent with this viewpoint, the gut microbiota in childhood is generally considered unstable and highly susceptible to environmental influences. Given the dramatic increases in the incidence of immune-mediated diseases in Western society and the strong association with altered microbial diversity [10], [48] it is important to consider that the microbiota of children in Western countries is adversely affected and limited by low microbial diversity in the environment. Recent evidence has emerged that children migrating from the developing world to the Western world take on the same susceptibility risk to IBD as the population of the adoptive country, unlike adult migrants [49]. Clearly, lifestyle and hygiene alter gut microbial diversity, but equally loss of important ancestral microbes from our environments may have important health consequences [50]. The current work strengthens the notion that optimal acquisition of the adult microbiota requires continuous microbial exposure, biodiverse environmental ecosystems and processes of selection, succession and stabilization in the context of the developing and maturing gut. Future work focusing on childhood microbiota development in diverse environments is important in defining the optimum microbiota and the natural successional patterns of the adult microbiota. This knowledge may provide greater insight into the importance of microbial biodiversity and reversal of current human disease trends.

## Supporting Information

Figure S1 **Individual 16S rRNA gene library rarefaction curves from outdoor isolator-reared animals (OIs;** ***N = 5*** **).** Rarefaction curves were generated by plotting the number of phylotypes (OTUs) against the number of clones sequenced. At 99% cut-off, rarefaction analysis suggested that the individual animals within the OIs group possessed a highly diverse mucosa-associated bacterial community.
(TIF)

Figure S2 **Individual 16S rRNA gene library rarefaction curves from indoor isolator-reared animals (InIs;** ***N = 5*** **).** Rarefaction curves were generated by plotting the number of phylotypes (OTUs) against the number of clones sequenced. At 99% cut-off, rarefaction analysis suggested that the individual animals within the InIs possessed a highly diverse mucosa-associated bacterial community.
(TIF)
