# Supplementary figures and images for: Establishment of Normal Gut Microbiota Is Compromised under Excessive Hygiene Conditions
Source: PLoS One. 2011 Dec 2;6(12):e28284. doi: 10.1371/journal.pone.0028284 (PMC3229561; doi:10.1371/journal.pone.0028284)

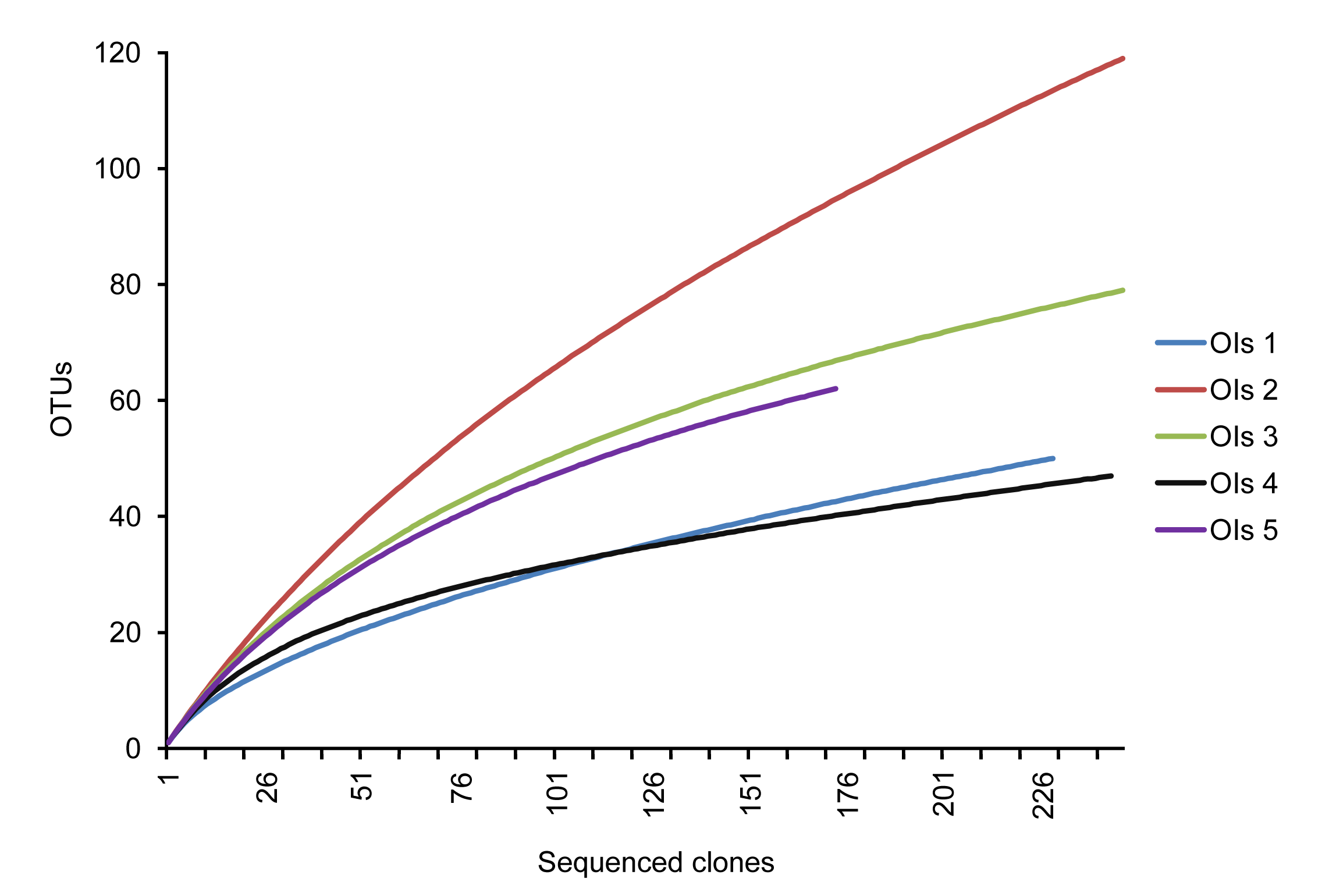

Supplement: Figure S1 — Individual 16S rRNA gene library rarefaction curves from outdoor isolator-reared animals (OIs; N = 5 ). Rarefaction curves were generated by plotting the number of phylotypes (OTUs) against the number of clones sequenced. At 99% cut-off, rarefaction analysis suggested that the individual animals within the OIs group possessed a highly diverse mucosa-associated bacterial community. (TIF) [file pone.0028284.s001.tif]

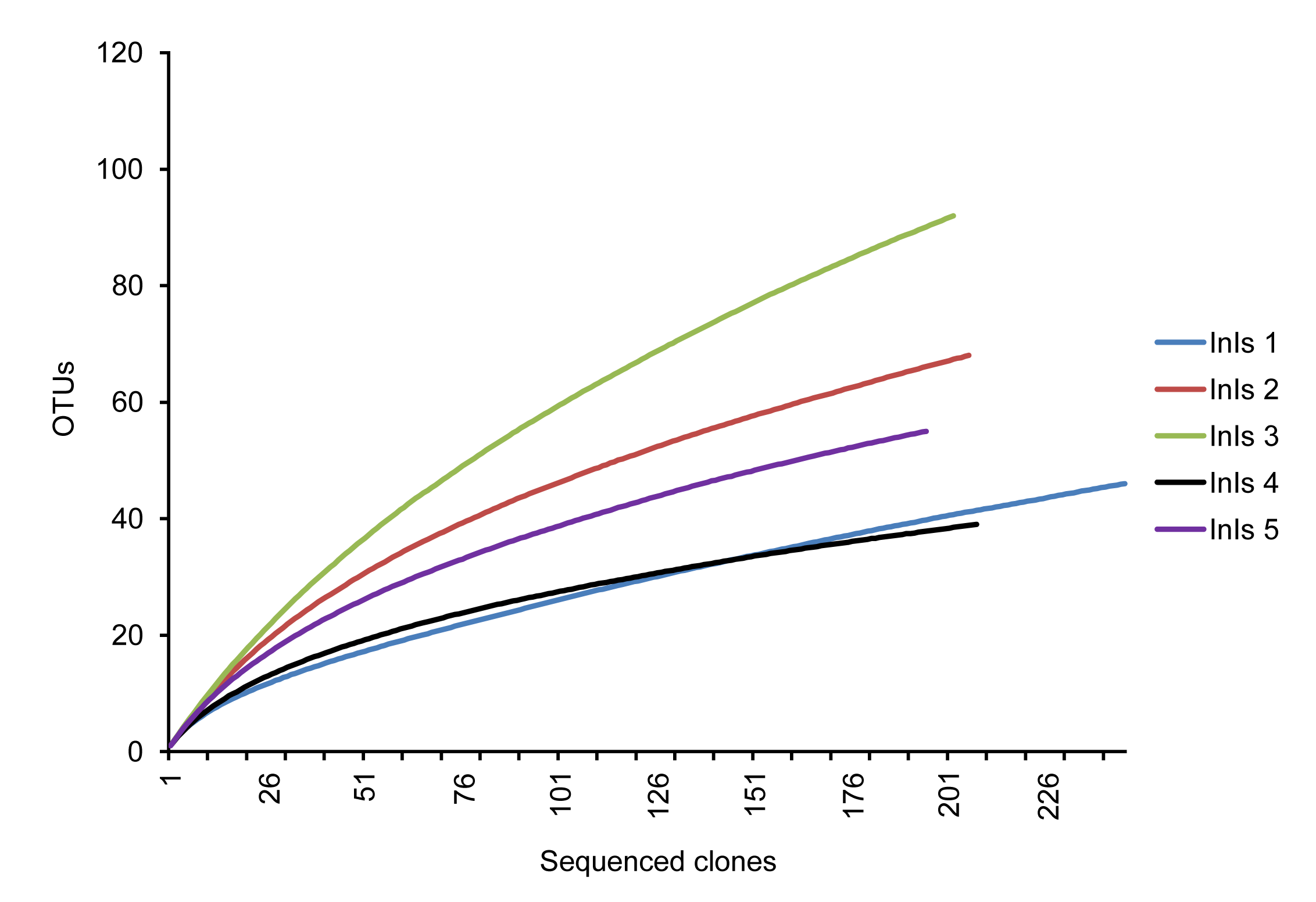

Supplement: Figure S2 — Individual 16S rRNA gene library rarefaction curves from indoor isolator-reared animals (InIs; N = 5 ). Rarefaction curves were generated by plotting the number of phylotypes (OTUs) against the number of clones sequenced. At 99% cut-off, rarefaction analysis suggested that the individual animals within the InIs possessed a highly diverse mucosa-associated bacterial community. (TIF) [file pone.0028284.s002.tif]
